# Fluxgate Sensor with Bifactor Excitation Mode

**DOI:** 10.3390/s23041775

**Published:** 2023-02-04

**Authors:** Ivan V. Bryakin, Igor V. Bochkarev, Vadim R. Khramshin, Vadim R. Gasiyarov

**Affiliations:** 1 Laboratory of Information and Measuring Systems, National Academy of Sciences of the Kyrgyz Republic, Bishkek 720010, Kyrgyzstan; 2 Department of Electromechanics, Kyrgyz State Technical University Named after I. Razzakov, Bishkek 720010, Kyrgyzstan; 3 Power Engineering and Automated Systems Institute, Nosov Magnitogorsk State Technical University, 455000 Magnitogorsk, Russia; 4 Department of Automation and Control, Moscow Polytechnic University, 107023 Moscow, Russia

**Keywords:** fluxgate converter, fluxgate sensor, electromagnetic field, electromagnetic acoustic effect, acoustic waves, variable magnetic field strength, eddy currents, permeability modulation

## Abstract

The paper considers non-destructive testing (NTDs) as a means to solve the flaw detection problems of magnetic products. It proposes a new probe-coil magnetic-field NDT, not requiring the pre-magnetization of the test object material, which is mandatory for all conventional magnetic flaw detection techniques. A new bifactor excitation of the fluxgate sensor’s sensitive element, based on double μ-transformation through the simultaneous activation of magnetic-modulating and electromagnetic-acoustic effects, is theoretically justified. The physical processes underlying the proposed technique are considered in detail, and a scheme for its practical implementation is described. The authors provide a variant of the new fluxgate’s original design, implementing the proposed excitation technique. The specifics of implementing the fluxgate operating modes are analyzed, testifying to the possibility of detecting a given class of flaws with the required coverage as well as ensuring the required diagnostic resolution during flaw detection, which, in fact, indicates a more reliable identification of both the flaw type and location. Herewith, the new fluxgate type features the advantages of improved functionality and lower cost due to its simple design. The paper also considers a method to experimentally study the capabilities of the proposed fluxgate sensor with a new bifactor excitation in detail. The results of the experimental study into its key specifications are provided, confirming its high resolution, narrower zone of uncertainty, and the possibility of detecting smaller flaws at greater depths compared to available analogs.

## 1. Introduction

Currently, non-destructive testing is mainly used to solve flaw detection problems. NDT forms the basis for controlling various products and working components, providing their safe and reliable operation without affecting their further use. NDT allows for the real-time monitoring of the tested object quality both in manufacture and in operation, thereby preventing early breakdown and failure. Unlike destructive testing, NDT procedures are applicable to 100% of products as they do not damage their material and structure and, therefore, do not affect their performance [1,2,3,4,5].

Various NDT techniques based on optical, electrical, magnetic, acoustic, and other properties of materials can be used to identify flaws or inhomogeneities, their nature, size, and location [6]. As most industrial equipment and production systems have components made of ferromagnetic structural materials such as steel or cast iron, the magnetic measurement-based NDT of their quality is most advisable [7,8,9,10,11]. We can specify the following examples of the most common and critical objects containing ferromagnetic components requiring periodic NDT during the entire service life without violating their functions: lifting mechanisms containing steel cables and ropes [12,13], power transmission lines based on, e.g., cables with steel-aluminum wires or ferromagnetic shields [14], steel or cast-iron pipelines [15], and various rolled metal products (sheet, section, shaped) [16,17].

Measuring instruments and systems, implementing this technique and design to read the physical parameters of the measurement object, include various types of converters. Thus, magnetic flaw detection uses fluxgate converters (FGCs) to detect subsurface flaws. Obviously, the significance and importance of such measurements determine a wide variety of magnetometry techniques and tools [18].

The fluxgate non-destructive testing technique is based on a fluxgate sensor detecting stray magnetic fields arising in the subsurface flaw area and is designed to detect cracks, holes, etc., in the tested object. The fluxgate sensor (FS) detects changes in the magnetic field strength over the flaw and converts the field strength gradient into an electrical signal. FS-based FGCs [19,20,21] are widely used here due to a lot of positive features. FSs have certain advantages compared to other sensors, i.e., small dimensions and low power consumption, high sensitivity and reliability, and resistance to external impacts. Therefore, the FS-based FGCs are continuously studied to improve and expand the performance of their key functional component—the FS [22,23,24,25,26,27]. Thus, the problem of improving the FS for precision FGCs is relevant, and research in this area is of great scientific, technical, and practical importance.

## 2. Analysis of Available Fluxgate Converters

The FGC is a quite complex electromagnetic system containing a ferromagnetic element (core) and one or more windings as basic working nodes, which perform the functions of exciting or measuring components. The cores are made of soft magnetic material—permalloy, and have a lot of structural modifications with different rod numbers and configurations, open and closed cores, spraying, etc.

The FGC’s sensitive element is a ferromagnetic core. The FGC operation is based on changing the magnetic state (i.e., the magnetic permeability µ) of a ferromagnetic core excited by a constant-intensity variable magnetic field under the impact of the measured magnetic field.

Passive induction converters are used to measure variable magnetic fields and characteristics. However, such converters do not allow measuring constant magnetic fields or characteristics when no EMF is induced in the converter circuit. In this case, active induction converters are used with appropriate excitation nodes to modulate the magnetic field.

FGCs may differ in the ferromagnetic core excitation way. Therefore, before studying the features of the developed FS excitation technique, let us briefly consider the available converter types.

**Figure 1:**
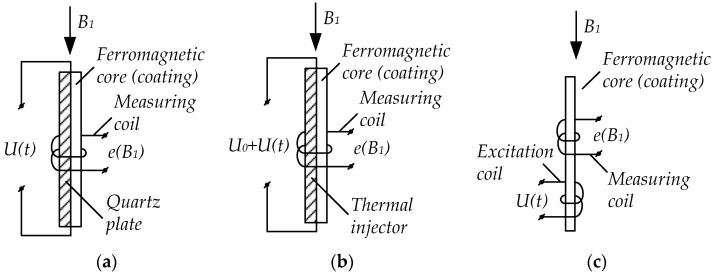
Fluxgate converters with mechanical (**a**), thermal (**b**), and magnetic (**c**) excitation.

There are three basic FGC excitation ways: 1—mechanical; 2—thermal; 3—magnetic. The schemes for constructing these FGCs are shown in Figure 1.

These excitation techniques are structurally implemented as follows. The first type of mechanical excitation of FGC’s electromagnetic system comprises a measuring coil with a quartz bar inside, having a thin permalloy film of a ferromagnetic coating (Figure 1a). This quartz bar performs the functions of a mechanical excitation element as when a resonant frequency voltage is applied to the bar plates, it periodically changes its linear dimensions and thereby affects the permalloy film. This causes a periodic change in its magnetic permeability. As a result, an EMF is induced in the measuring coil, directly proportional to the measured field induction *B_i_*. However, despite the obviously simple implementation, this converter excitation technique has low sensitivity as the magnetic permeability variation range is negligible.

The second type of thermal excitation of FGCs contains a thermal injector with small thermal inertia as an excitation element. It is in direct contact with a ferromagnetic element—a thin ferromagnetic plate made of a material with a low Curie point (Figure 1b). The injector with a ferromagnetic element is located inside the measuring coil. It operates as follows. The injector simultaneously passes direct and alternating currents. The DC magnitude is chosen to heat the ferromagnetic plate to a temperature close to the Curie point. Its magnetic permeability, therefore, grows sharply as the ferromagnetic domain friction decreases, facilitating their rotation under the external magnetic field (the Hopkinson effect). Alternating current ensures the plate temperature pulsation near the Curie point with a doubled frequency. The ferromagnetic plate’s permeability also pulsates proportionally to the temperature pulsation. This induces an EMF signal in the measuring coil, proportional to the external measured magnetic field.

As the permeability pulsations near the Curie point due to the thermal modulation have a significant modulation index, the thermal excitation FGC has a higher sensitivity than the mechanical excitation one and can be used to measure weak magnetic fields.

The third type of magnetic excitation of FGCs called fluxgates implements the nonlinear dependence *μ* = *f*(*H*), typical of ferromagnetic materials. Their design comprises a rod made of magnetically soft ferromagnetic material with excitation and measuring coils placed on it (Figure 1c). Such a converter’s operation is based on recording periodic changes in the measured magnetic flux through a ferromagnetic core with the permeability changed periodically under the excitation coil field. Thereat, an alternating current is passed through the excitation coil with a magnitude facilitating reversal magnetization of the core up to saturation under the variable magnetic field inside. The changing magnetic flux induces an EMF in the measuring coil. Thereat, in the absence of an external measured field, the EMF changes according to a harmonic law, and when the field appears, the EMF magnitude and harmonic composition change and even harmonics occur with a magnitude directly proportional to the measured field intensity. This generates a data signal in the form of a voltage at the measuring coil terminals, which contains a range of measured magnetic field parameters: amplitude, frequency, and the intensity vector direction. These properties are the fluxgate advantages compared to FGCs employing other excitation ways.

The following equation for the EMF induced in the measuring winding is true for all three FGC types according to the electromagnetic induction law [28,29]:

(1) $$e=-w\times s_{i}\times i_{w}^{0}\times \frac{d}{dt}B(t)=-w\times s_{i}\times i_{w}^{0}\times \frac{d}{dt}[{\hat{\mu }}^{*}(t)\times H_{0}]$$

where $i_{w}^{0}$ is the unit vector coinciding with the winding turn plane; *w* is the measuring winding turn number; *s_i_* is the core cross-sectional area in the $i_{w}^{0}$ direction; ${\hat{\mu }}^{*}(t)$ is the relative permeability tensor of the core material; **B** and **H**_0_ are the measured magnetic field induction and strength vectors, respectively.

## 3. Study Results

Summarizing the features of the considered magnetic NDT techniques, one can note that each of these well-known methods is based on a single physical effect determining, in fact, both the technique opportunities and implementation specifics.

The present paper proposes an *FS* version with a new principle of bifactor excitation based on electromagnetic acoustic and magnetic modulation (EMA and MM) effects. This FS also allows the implementation of the NDT method integrating the potential of three magnetic NDT techniques: constant magnetic field, variable magnetic field, and the fluxgate technique. It does not require a mandatory pre-magnetization of the test object material.

In this case, a constant magnetic field is used as a probing physical one modulated by a variable magnetic field through a μ-modulation, and this process variability depends on the structural conditions of the test object material.

**Figure 2:**
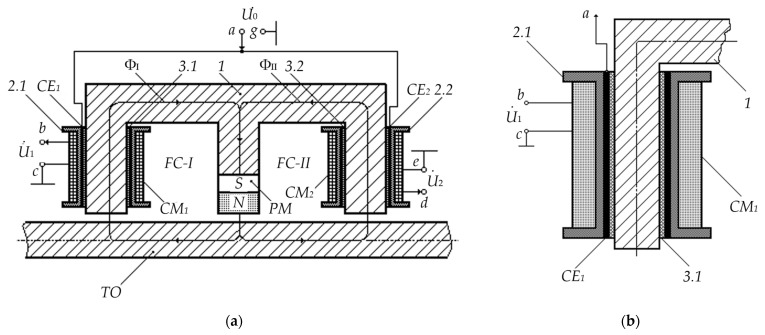
The FS Design Solution Version (**a**) and Increased Fragment of the Left Side of the Cylindrical Electrode (**b**).

Figure 2 provides a version of the new FS design solution.

The FS key structural and functional elements are: 1—E-core made of an electrically conductive ferrimagnet; TO—tested object—long ferromagnetic element in the form of a steel bar; PM—permanent magnet; MC_1_ and MC_2_—electric windings of the first and second measuring coils, respectively; 2.1 and 2.2—dielectric formers of M*C*_1_ and MC_2_, respectively; CE_1_ and CE_2_—the first and second cylindrical electrodes, which are excitation elements for MC_1_ and MC_2_, respectively, made of electrically conductive material (copper, aluminum, etc.) in the form of thin-walled tubes with a slot along the generatrix, located coaxially on the inner surface of dielectric formers 2.1 and 2.2, respectively; 3.1 and 3.2—dielectric cylinder bushings located locally in particular core sections; ‘*a*’—terminal to connect the excitation voltage ${\dot{U}}_{0}$ relative to the ‘case’; ‘*b*’ and ‘*d*’—terminals to connect output signals ${\dot{U}}_{1}$ and ${\dot{U}}_{2}$ of, respectively, MC_1_ and MC_2_ relative to the ‘case’, which are starts of electrical windings CE_1_ and CE_2_, respectively; ‘*c*’ and ‘*e*’—terminals for connecting the ‘ends’ of corresponding electric windings CE_1_ and CE_2_ to the high-frequency generator ‘case’; ‘g’—the ‘case’ of the frequency generator.

A feature of this FS type is the use of cognominal terminals of electrical windings (the winding starts) as output signals MC_1_ and MC_2_ (relative to the ‘case’), which, in turn, is among the major conditions for the normal operation of the considered FS. In addition, CE_1_ and CE_2_ with lower wire layers of, respectively, windings of MC_1_ and MC_2_ form coupling capacitors $C_{coup}^{\prime }$ and $C_{coup}^{\prime \prime }$. The excitation voltage ${\dot{U}}_{0}$ relative to the ‘case’ is supplied through the coupling capacitors $C_{coup}^{\prime }$ and $C_{coup}^{\prime \prime }$, respectively, to the starts of electrical windings MC_1_ and MC_2_, coherently spatially arranged (start-end) on the core.

The E-core forms with the TO two interconnected ferrimagnetic circuits (FC) I and II, through which the magnetic fluxes Φ_I_ and Φ_II_ are configured in a certain way, initiated by combined physical fields: the PM constant magnetic field and variable magnetic fields formed by excitation currents of electric windings MC_1_ and MC_2_.

When a high-frequency generator supplies an electric potential φ_e_ relative to the case’s electric potential φ_0_ (grounding point) to CE_1_ and CE_2_, respective electric potentials φ′_e_ and φ″_e_ are induced in the lower wire layers of windings of MC_1_ and MC_2_. Moreover, the symmetrical spatial arrangement of passage coils and their identical design parameters allow the assumption that φ′_e_ = φ″_e_ = φ_e_. In this case, the required symmetry in the spatial arrangement of passage coils and the balanced lower level of the zero signal are ensured by the corresponding axial shift of passage coil sections relative to the constant magnet. Then, in the series electrical resonance mode (at the cyclic frequency ω_r_), currents in electric windings of MC_1_ and MC_2_ are determined by the equation:

(2) $$i_{1}(t)=i_{2}(t)=i(t)=(\varphi_{e}-\varphi_{0})/R=u_{0}(t)/R=(U_{0m}\times \cos\omega_{p}t)/R$$

where φ_0_ = 0; *R* = *R*_1_ = *R*_2_, and *R*_1_ and *R*_2_ are active resistances of the coil windings (MC_1_ and MC_2_), respectively; *U*_0*m*_ is the high-frequency generator electrical voltage amplitude.

In this case, the strength of the variable magnetic field formed by MC_1_ and MC_2_ excitation currents can be expressed as follows [30]:

(3) $$H_{1}(t)=H_{2}(t)=H(t)=\frac{2\times \sqrt{2}\times w\times U_{0m}}{l\times R}\times \cos\omega_{p}t=H_{m}\times \cos\omega_{p}t,$$

where *l* = *l*_1_ = *l*_2_ is the length of electric windings of MC_1_ and MC_2_; *w* = *w*_1_ = *w*_2_ is the number of turns of electric windings of MC_1_ and MC_2_; $H_{m}=\frac{2\times \sqrt{2}\times w\times U_{0m}}{l\times R}$ is the strength amplitude of the magnetic field produced by the current *i*(t).

Consider the issues relating to the peculiarities of magnetic fluxes Φ_I_ and Φ_II_ in the magnetic cores corresponding to FCs.

According to the electrical laws, magnetic fluxes Φ_I_ and Φ_II_ generated in FC-I and FC-II can be expressed, respectively:

(4) $$\begin{array}{l} \Phi_{I}=\frac{F_{I}}{R_{I_{M}}^{\prime }+R_{I_{M}}^{\prime \prime }+R_{I_{M}}^{\prime \prime \prime }}=\frac{F_{I}}{R_{I_{M}}^{*}+R_{I_{M}}^{\prime \prime \prime }};\\ \Phi_{II}=\frac{F_{II}}{R_{{\mathrm{II}}_{M}}^{\prime }+R_{{\mathrm{II}}_{M}}^{\prime \prime }+R_{{\mathrm{II}}_{M}}^{\prime \prime \prime }}=\frac{F_{II}}{R_{II_{M}}^{*}+R_{I_{M}}^{\prime \prime \prime }}, \end{array}$$

where $F_{I}=F_{I}^{=}+F_{I}^{\sim }$ is the total magnetizing force in FC-I, and $F_{I}^{=}\;\mathrm{and}\;F_{I}^{\sim }$ are magnetizing forces created in FC-I by, respectively, *H*_0_ and *H*(t); $F_{II}=F_{II}^{=}+F_{II}^{\sim }$ is the total magnetizing force in FC-II, and $F_{II}^{=}\;\mathrm{and}\;F_{II}^{\sim }$ are magnetizing forces created in FC-II by, respectively, *H*_0_ and *H*(t); $R_{I_{M}}^{\prime \prime \prime }$ is the magnetic resistance of the TO section located in the working area of FC-I and is its controlled element; $R_{I_{M}}^{*}=R_{I_{M}}^{\prime }+R_{I_{M}}^{\prime \prime }$ is the total magnetic resistance of FC-I structural elements; $R_{I_{M}}^{\prime }\;\mathrm{and}\;R_{I_{M}}^{\prime \prime }$ are magnetic resistances of, respectively, FC-I ferromagnetic and dielectric (air gap) structural elements; $R_{{\mathrm{II}}_{M}}^{\prime \prime \prime }$ is the magnetic resistance of the TO section located in the working area of FC-II and is its controlled element; $R_{II_{M}}^{*}=R_{{\mathrm{II}}_{M}}^{\prime }+R_{{\mathrm{II}}_{M}}^{\prime \prime }$ is the total magnetic resistance of FC-II structural elements, and $R_{{\mathrm{II}}_{M}}^{\prime }\mathrm{and}\;R_{{\mathrm{II}}_{M}}^{\prime \prime }$ are magnetic resistances of, respectively, FC-II ferromagnetic and dielectric (air gap) structural elements.

Note that $R_{I_{M}}^{*}=R_{{\mathrm{II}}_{M}}^{*}=R_{M}^{*}=const\;$as they are the parameters of identical structural elements of FC-I and FC-II; $R_{I_{M}}^{\prime \prime \prime }$ and $R_{{\mathrm{II}}_{M}}^{\prime \prime \prime }$ are variable parameters of controlled elements of, respectively, FC-I and FC-II, corresponding to the condition $R_{I_{M}}^{\prime \prime \prime }=R_{{\mathrm{II}}_{M}}^{\prime \prime \prime }=R_{M}^{\prime \prime \prime }$ in the absence of a flaw in the working areas of FC-I and FC-II. Moreover, it is considered that $R_{I_{M}}^{\prime \prime \prime }$ ≫ $R_{M}^{*}$ and $R_{{\mathrm{II}}_{M}}^{\prime \prime \prime }$ ≫ $R_{M}^{*}$.

Considering the aforementioned, after simple conversions (4), we obtain:

(5) $$\begin{array}{l} \Phi_{I}=\frac{F_{I}}{\left(1+\frac{G_{I_{M}}^{\prime \prime \prime }}{G_{I_{M}}^{*}}\right)}\times G_{I_{M}}^{\prime \prime \prime }=F_{I}\times G_{I_{M}}^{\prime \prime \prime }=F_{I}\times \mu_{I_{M}}^{\prime \prime \prime }\times \frac{l_{I_{M}}^{\prime \prime \prime }}{s_{I_{M}}^{\prime \prime \prime }};\\ \Phi_{II}=\frac{F_{II}}{\left(1+\frac{G_{{\mathrm{II}}_{M}}^{\prime \prime \prime }}{G_{II_{M}}^{*}}\right)}\times G_{{\mathrm{II}}_{M}}^{\prime \prime \prime }=F_{II}\times G_{{\mathrm{II}}_{M}}^{\prime \prime \prime }=F_{II}\times \mu_{{\mathrm{II}}_{M}}^{\prime \prime \prime }\times \frac{l_{{\mathrm{II}}_{M}}^{\prime \prime \prime }}{s_{{\mathrm{II}}_{M}}^{\prime \prime \prime }}, \end{array}$$

where $G_{I_{M}}^{\prime \prime \prime }=1/R_{I_{M}}^{\prime \prime \prime }=\mu_{I_{M}}^{\prime \prime \prime }\times \frac{l_{I_{M}}^{\prime \prime \prime }}{s_{I_{M}}^{\prime \prime \prime }}$ and $G_{{\mathrm{II}}_{M}}^{\prime \prime \prime }=1/R_{{\mathrm{II}}_{M}}^{\prime \prime \prime }=\mu_{{\mathrm{II}}_{M}}^{\prime \prime \prime }\times \frac{l_{{\mathrm{II}}_{M}}^{\prime \prime \prime }}{s_{{\mathrm{II}}_{M}}^{\prime \prime \prime }}$ are magnetic conductivities of the TO sections located in the working areas of FC-I and FC-II, respectively; $\mu_{I_{M}}^{\prime \prime \prime }$, $l_{I_{M}}^{\prime \prime \prime }$, and $s_{I_{M}}^{\prime \prime \prime }$ are, respectively, the magnetic permeability, length, and cross-sectional area of the TO section located in the working area of FC-I; $\mu_{{\mathrm{II}}_{M}}^{\prime \prime \prime }$, $l_{{\mathrm{II}}_{M}}^{\prime \prime \prime }$, and $s_{{\mathrm{II}}_{M}}^{\prime \prime \prime }$ are, respectively, the magnetic permeability, length, and cross-sectional area of the TO section located in the working area of FC-II; $G_{I_{M}}^{*}=1/R_{I_{M}}^{*}$ and $G_{II_{M}}^{*}=1/R_{II_{M}}^{*}$ are total magnetic conductivities of structural elements of FC-I and FC-II, respectively; $\frac{G_{I_{M}}^{\prime \prime \prime }}{G_{I_{M}}^{*}}<<1$ and $\frac{G_{{\mathrm{II}}_{M}}^{\prime \prime \prime }}{G_{{\mathrm{II}}_{M}}^{*}}<<1$.

The analysis of Equation (5) allows for drawing an important conclusion that the time invariability of magnetic fluxes Φ_I_ and Φ_II_ parameters is mainly determined by the variability of parameters $\mu_{I_{M}}^{\prime \prime \prime }$ and $\mu_{{\mathrm{II}}_{M}}^{\prime \prime \prime }$.

Further, consider the impact of combined magnetic fields (constant and variable) in the FS core directly on the ferrimagnetic circuits’ conductive ferrimagnetic structural element material structure.

When studying the magnetic permeability μ as a multi-affected particular parameter of the core material, variable magnetic fields excited by MC_1_ and MC_2_ and initiating the MM effect can be considered as the major impact and an acoustic field, which is a manifestation of the EMA effect, as the additional one. Therefore, the proposed FS version is, in fact, a parametric modulator, where the measured parameter (the core’s constant magnetic field) is modulated due to a bifactor impact on a particular parameter, i.e., the μ-modulation is simultaneously determined by the MM and EMA effects.

MM involves variating the ferromagnetic conductive material magnetic state with simultaneous magnetization in variable and measured constant magnetic fields. Modulation by such a total magnetic flux is possible due to the non-linear magnetic circuit properties, and the processes occurring in it are always associated with the interaction of at least two magnetic fields—an external measurable one and a variable excitation field induced, e.g., by an electric current in the excitation coil.

As for the mode of the rod ferromagnetic system material magnetic permeability conversion (μ-modulation) due to the MM, one can state that, in fact, it is the operation mode of a fluxgate with longitudinal excitation. Therefore, according to the existing parametric theory of fluxgates with longitudinal excitation:

(6) $$B_{MM}(t)=\mu_{0}\times \mu^{*}[H(t)]\times H_{0}=\mu_{0}\times \mu_{MM}^{*}(t)\times H_{0}$$

where μ_0_ = 4π × 10^−7^ H/m is the magnetic constant (magnetic permeability of vacuum); *H*(*t*) = *H_m_* × sin *ωt* is the exciting magnetic field strength, *H_m_* is the *H*_1_ variable magnetic field amplitude; ω is the cyclic frequency; *H*_0_ is the constant magnetic field strength; $\mu^{*}[H(t)]$ is the function describing the law of change in the magnetic permeability of the ferromagnetic core material under the exciting magnetic field, which can be considered as the time function $\mu_{MM}^{*}(t)$ for the given exciting magnetic field magnitude.

Note that the physical nature of the acoustic wave electromagnetic generation and reception phenomena is quite complex. Therefore, to better understand the essence of the proposed technical solution, consider the used EMA conversion effect in more detail.

It has been established that within a wide range of frequencies, magnetic fields, and temperatures, various mechanisms of contactless electromagnetic and acoustic waves conversion at the interface of metallic materials can be traced, united by the common concept of EMA conversion [31]. Such a conversion essence is that in a medium with neither piezoelectric nor magnetostrictive properties, an incident electromagnetic wave excites ultrasonic waves of the same or multiple frequency. It should be emphasized that the existence of an interface as the excitation source concentration location is of fundamental significance.

Direct and reverse EMA conversion is the conversion of electromagnetic waves into elastic ones and vice versa, respectively. Moreover, the ‘electromagnetic field–elastic oscillations–electromagnetic field’ conversion can be considered a double EMA conversion.

The opposite effect, i.e., receiving acoustic signals using the EMA conversion, takes place due to the EMF arising in the coil winding affected by electromagnetic radiation of free electrons of the FC-I and FC-II material under the action of acoustic waves. In this case, according to the reciprocity theorem, the acoustic characteristics of EMA converters during generation and reception are identical. For example, the selectivity of waves and radiation patterns of EMA converters correspond to those when they work as receivers.

In fact, the EMA conversion is based on the phenomenon of mutual conversion of elastic and electromagnetic fields. The conversion of fields in solids is possible due to many physical phenomena responsible for, e.g., magnetostriction, the Lorentz force, or the force determined by the magnetization gradient. In this case, the equations of electrodynamics describing the Lorentz forces and magnetization are introduced and used along with the standard theory of elasticity to describe the generation and reception of waves, and magnetostriction is included in the model using the corresponding equations relating the elastic field to the electromagnetic field.

Summarizing the aforementioned, one can state that the EMA excitation and reception of ultrasonic oscillations are based on three effects of the electromagnetic field interaction with the structural components of the affected object material:

1. Magnetostriction—a physical effect when a variable external magnetic field changes the ferromagnetic material dimensions. The opposite effect is magnetoelasticity.
2. Magnetic interaction occurs when a ferromagnetic material and an AC conductor mutually attract and repel. The coil repulsion and attraction have a reverse mechanical impact on the test object, in which elastic oscillations arise.
3. Electrodynamic interaction involves the excitation of eddy currents in the conductive material, interacting with a constant magnetic field and causing oscillations, which, in turn, leads to the oscillation of atoms, i.e., the material crystal lattice (mechanical stresses occur, further causing elastic acoustic oscillations).

The operation of the proposed FS version, determined primarily by the physical properties of the core material, is based on the electrodynamic interaction. In this case, the ponderomotive forces arising when eddy currents interact with the primary field and the magnetic ponderomotive forces arising when the primary field interacts with a ferromagnetic substance are directed oppositely and balanced for some parameter magnitudes. Thereat, ferromagnet subsystems of various physical natures such as electrical, magnetic, magnetoelastic, and elastic ones are involved in the conversion, which ultimately explains the high EMA conversion sensitivity to various variations in the modulated constant magnetic field.

We study the essence of the primary electromagnetic field interaction with the secondary one and the conversion of the electromagnetic field energy into the acoustic field one in more detail.

When placing an electromagnetic field source, e.g., a solenoid winding with electric current, at the surface of an arbitrary conductive medium inside that solenoid, each electron in it experiences the action of the corresponding force under such a field, and all electrons inside the Fermi sphere receive the corresponding acceleration in the conductive medium. Such a directed motion of valence electrons causes charge transfer in a solid body, i.e., an eddy current.

Note that eddy currents induced by an electromagnetic field source in a conductive medium reflect energy back to the solenoid winding, and according to the conventional view, charge carriers distribute inside a conductive medium from the upper- to the lower-surface magnitude with a sharp frequency dependence, obeying the exponential law. Thereat, the electrons moving near the conductive medium surface and contributing to the eddy current are affected by the solenoid magnetic field (Lorentz force) pushing the electrons away from the surface. In other words, the carriers forming the eddy current move deep into the medium from the surface under the impact of Lorentz forces, creating a charge-carrier-free zone near the medium surface.

In this case, the essence of the phenomenon of electromagnetic sound generation in a conductive medium is that the converter’s (excitation source) variable electromagnetic field interacts with the conductive medium’s electronic system. The perturbation of electrons under the external electromagnetic impact, in turn, causes the elastic medium motion due to the electron–lattice interaction, and the perturbation propagates deep into the medium in the form of acoustic waves.

A change in the linear or volumetric elementary volume dimensions under an electromagnetic field is determined by the magnetostrictive interaction.

The interaction of eddy currents with the bias field induction *B*_0_, causing the occurrence of *F*_A_ (Ampere force), is defined by acoustic oscillations taking place in the electrodynamic mechanism [28,29]:

*F*_A_ = *i_e_* × *B*_0_ × *dl* (7)

where *i_e_* is the eddy current of a section with the *dl* length.

Elastic forces arise in the TO near-surface layer determined by the skin layer depth δ:

(8) $$\delta =\sqrt{2/(\omega \times \mu_{0}\times \mu \times \sigma )}$$

where μ_0_ = 4π × 10^7^ H/m; μ is the relative magnetic permeability; σ is the electrical conductivity; ω is the circular oscillation frequency.

The EMA converter considered is characterized by the simultaneous emission of elastic waves from each point of the surface of the core’s FC-I and FC-II structural elements, located, respectively, under MC_1_ and MC_2_. Thus, waves propagate in the object cross-section in all radial directions.

**Figure 3:**
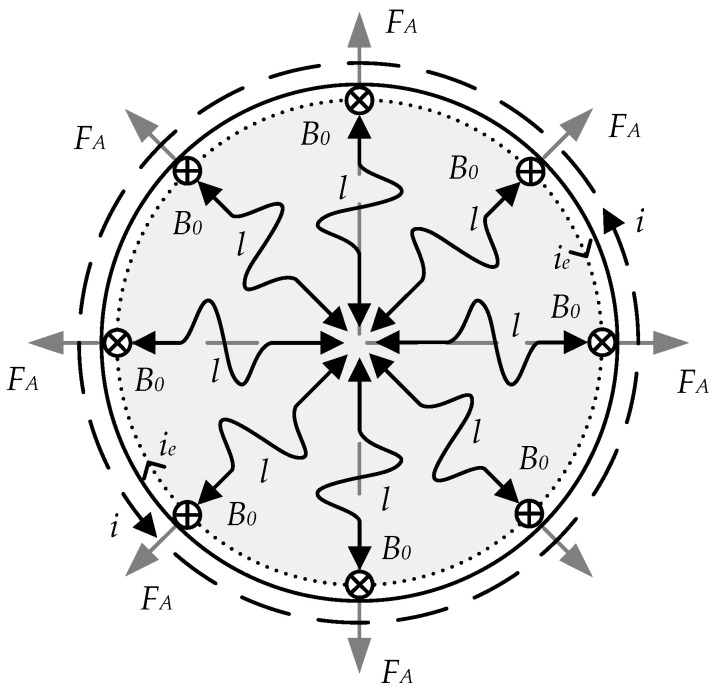
Model of the Radial Longitudinal Wave (L-Wave) Propagation over the Cylinder Cross-Section.

The process of longitudinal *L*-waves propagation over the cylindrical core cross-section based upon the electrodynamic interaction mechanism is shown in Figure 3.

The predominant excitation of a single wave type is determined by the mutual orientation of the bias field with induction *B_0_* and eddy currents *i_e_* flowing along the core element perimeter.

When both longitudinal- and transverse-plane monochromatic acoustic waves are excited, the equation of forced acoustic oscillations propagating from the interface can be written as follows [30,31]:

(9) $$\frac{\partial^{2}\zeta }{\partial t^{2}}-v^{2}\times \frac{\partial^{2}\zeta }{\partial z^{2}}=\frac{1}{\rho \times c}\times [j\times H_{0}]$$

where **H**_0_ is the PM field strength vector magnitude; **j** is the skin layer AC density vector value; ζ is the displacement vector; *v* is the acoustic wave speed in the ferrite rod material; ρ is the ferrite rod material specific density; *c* is the speed of light.

Assuming that the variable magnetic (Emicon excitation) field changes according to the law exp [*i* × (ω*t* − *k* × *z*)], the skin layer AC density can be expressed as follows:

(10) $$j(z,\;t)=\frac{(1+i)\times c}{4\pi \delta }\times H_{m}\times \exp\left[-(1+i)\times \frac{z}{\delta }\right]\times e^{i\omega t}$$

where *H_m_* is the variable magnetic field amplitude; $\delta =c\times \sqrt{2\pi \omega \sigma }$ is the metal conductivity; ω is the cyclic frequency.

At the distances exceeding the skin layer thickness, considering (8), the solution for Equation (9) will take the form:

(11) $$\zeta_{m}=\frac{H_{0}\times H_{m}}{4\pi \times \rho \times v\times \omega }\times \frac{1}{\sqrt{1+\beta^{2}}}$$

where β = *q*^2^ × δ^2^/2, *q* = 2π/λ, and λ is the acoustic wavelength.

In magnetically ordered media, the acoustic wave propagation is associated with the conversion of waves at domain walls and the excitation of coupled magnetoelastic oscillations *q*(*k_n_*,*t*) accompanied by magnetization ones:

Δ*J_q_*(*k_n_*,*t*) = λ × *q*(*k_n_*,*t*) (12)

where λ is a coefficient depending on the magnetoelastic tensor magnitude, the wave vector *k_n_*, and the difference between the spin and elastic wave frequencies.

Considering (11) and (12), as well as based on the fact that the magnetic susceptibility χ = *J*/H and μ = 1 + χ, one can write:

(13) $$\mu =f[\zeta (t)]=\mu^{*}[\zeta (t)]$$

Therefore, similarly to (6) and according to (12) and (13), the following will be true for the μ-modulation mode due to the EMA effect:

(14) $$B_{AM}(t)=\mu_{0}\times \mu^{*}[\zeta (t)]\times H_{0}=\mu_{0}\times \mu_{AM}^{*}(t)\times H_{0}$$

where ζ(t) is the displacement function of structural components of the material of the rod ferromagnetic system’s 1′ and 1″ elements; $\mu_{AM}^{*}[\varsigma (t)]$ is the function describing the law of change in the magnetic permeability of the material of the rod ferromagnetic system’s 1′ and 1″ elements under an exciting acoustic field, which, at a given excitation field amplitude, can be considered as the time function $\mu_{AM}^{*}(t)$.

Considering the joint modulating impact of acoustic and variable magnetic fields on the magnetic permeability of the core’s FC-I and FC-II element material, write the following equation:

(15) $$\mu^{*}=f[H(t);\zeta (t)]$$

Note that for the considered physical processes, modulation is understood as a change in the state of the magnetic permeability of the core’s FC-I and FC-II element material when exposed to physical fields.

According to the existing parametric theory of fluxgates with longitudinal excitation, applying the small-impact Taylor series expansion of *B*(*H*_Σ_) at *H*_Σ_ = *H*(t) + *H*_0_, and considering that for our case, the function $\mu^{*}=f[H(t);\zeta (t)]$ is even, we can write:

(16) $$\mu^{*}(t)=\mu_{H}\times [1+(m_{MM}+m_{AM})]\times \cos(2\omega_{p}t)$$

where $m_{AM}=\mu_{m}\times \eta_{AΜ}\times \zeta_{m}\times \mu_{H}^{-1}$ and $m_{MM}=\mu_{m}\times \eta_{MM}\times H_{m}\times \mu_{H}^{-1}$ are the depths of, respectively, acoustic and magnetic modulation; η*_AM_* and η*_MM_* are factors of, respectively, the acoustic magnetic and magnetic modulation conversions; ω_P_ is the conversion excitation cyclic frequency at coincident frequencies of electromechanical and magnetic resonances.

From Equations (15) and (16), it follows that the PM constant magnetic field *H*_0_(t) directed axially to FC_1_ and FC_2_ is converted into a variable magnetic field with the corresponding induction by the oscillating magnetic permeability of the ferromagnetic material of FC_1_ and FC*_2_* due to parametric modulation:

(17) $$\begin{array}{l} B_{I}(t)=\mu_{0}\times \mu_{I}^{*}(t)\times H_{0}=\mu_{0}\times \mu_{I}{}_{n}\times [1+(m_{MM}+m_{AM})\times \cos(2\omega_{p}t)]\times H_{0};\\ B_{II}(t)=\mu_{0}\times \mu_{II}^{*}(t)\times H_{0}=\mu_{0}\times \mu_{II}{}_{n}\times [1+(m_{MM}+m_{AM})\times \cos(2\omega_{p}t)]\times H_{0}. \end{array}$$

Variations in the PM magnetic field, caused now by modulating processes of magnetic permeability, affect, respectively, the MC_1_ and MC_2_ windings, inducing the corresponding EMF in them:

(18) $$e_{1}(t)=-s_{1}\times w\times \frac{dB_{I}(t)}{dt}\;\mathrm{and}\;e_{2}(t)=-s_{2}\times w\times \frac{dB_{II}(t)}{dt}.$$

Substituting (17) into (18), for each of the measuring coils, we finally obtain:

− for MC_1_

(19) $$\begin{array}{l} e_{1}(t)=-s_{1}\times w\times \mu_{0}\times \left[\mu_{I}^{*}(t)\times \frac{dH_{0}}{dt}+H_{0}\times \frac{d\mu_{I}^{*}}{dt}\right]=-s_{1}\times w\times \mu_{0}\times H_{0}\times \frac{d\mu^{*}}{dt}=\\ \;=2\times s_{1}\times w\times \mu_{0}\times \mu_{\mathrm{In}}\times \omega_{p}\times H_{0}\times (m_{MM}+m_{AM})\times \sin(2\omega_{p}t); \end{array}$$

− for MC_2_

(20) $$\begin{array}{l} e_{2}(t)=-s_{2}\times w\times \mu_{0}\times \left[\mu^{*}(t)\times \frac{dH_{0}}{dt}+H_{0}(t)\times \frac{d\mu_{II}^{*}}{dt}\right]=-s_{2}\times w\times \mu_{0}\times H_{0}\times \frac{d\mu_{II}^{*}}{dt}=\\ \;=2\times s_{2}\times w\times \mu_{0}\times \mu_{IIn}\times \omega_{p}\times H_{0}\times (m_{MM}+m_{AM})\times \sin(2\omega_{p}t). \end{array}$$

Further, based on (19) and (20), for the difference data signal from MC_1_ and MC_2_ relative to the ‘case’, we obtain:

(21) $$\begin{array}{l} \Delta e(t)=-s_{2}\times w\times \mu_{0}\times \left[\mu^{*}(t)\times \frac{dH_{0}}{dt}+H_{0}(t)\times \frac{d\mu_{II}^{*}}{dt}\right]=-s_{2}\times w\times \mu_{0}\times H_{0}\times \frac{d\mu_{II}^{*}}{dt}=\\ \;=2\times s_{2}\times w\times \mu_{0}\times \omega_{p}\times (m_{MM}+m_{AM})\times (\mu_{IH}-\mu_{IIn})\times H_{0}\times \sin(2\omega_{p}t). \end{array}$$

Considering that $e_{1}(t)=-w\times \frac{d\Phi_{I}(t)}{dt}$ and $e_{2}(t)=-w\times \frac{d\Phi_{II}(t)}{dt}$, as well as the results of analysis (5), Equation (21) can be transformed as follows:

(22) $$\begin{array}{l} \Delta e(t)=2\times s_{2}\times w\times \mu_{0}\times \omega_{p}\times (m_{MM}+m_{AM})\times (\mu_{I_{M}}^{\prime \prime \prime }-\mu_{{\mathrm{II}}_{M}}^{\prime \prime \prime })\times H_{0}\times \sin2\omega_{p}t=\\ \;=2\times s_{2}\times w\times \mu_{0}\times \omega_{p}\times (m_{MM}+m_{AM})\times \Delta \mu_{M}^{\prime \prime \prime }\times H_{0}\times \sin2\omega_{p}t. \end{array}$$

The resulting analytical expression (22) shows a clear implementation of the superposition principle in the form of a bifactorial additive modulating impact of two manifested basic physical effects initiated by the relevant activating physical fields, which, in turn, indicates an improved efficiency of the μ-modulation in general and the process variability depending on the availability of a flaw manifested in the parameter $\Delta \mu_{M}^{\prime \prime \prime }$.

## 4. Key FS Operating Modes

In summary, one can state that the specifics of the described FS version are the availability of five combined operating modes: 1—the bridge inductive-capacitive voltage divider mode; 2—the inductor mode, where MC_1_ and MC_2_ measuring coils, along with their direct purpose, function additionally as the elements generating an exciting variable magnetic field; 3—the mode of μ-modulation due to the MM effect; 4—the EMA-converter mode, implementing the occurrence of spatially periodic acoustic waves; 5—the mode of μ-modulation due to the EMA effect.

Consider simplistically the major specifics of the aforementioned FS operating modes.

**Figure 4:**
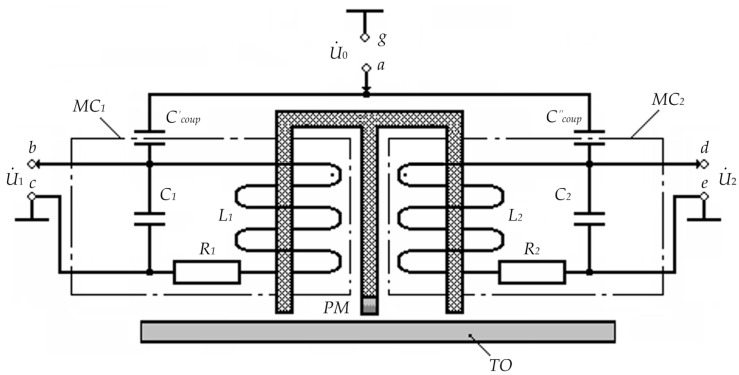
Circuit Diagram of the FS Substitution and Inclusion for the First Mode.

1. The bridge inductive-capacitive voltage divider mode. Figure 4 shows the circuit diagram of the substitution and inclusion of the considered FS version for the considered operating mode.

In Figure 4: ${\dot{U}}_{0}$ is the complex harmonic supply voltage of measuring coils; PM is the constant magnet inducing longitudinal magnetic fields in the core’s FC_1_ and FC_2_ elements with induction B_0_; C_1_ and C_2_ are inter-turn capacitances of, respectively, MC_1_ and MC_2_ windings; R_1_ and R_2_ are active resistances of, respectively, MC_1_ and MC_2_ windings; L_1_ and L_2_ are inductances of, respectively, MC_1_ and MC_2_ windings; $C_{coup}^{\prime }\;\mathrm{and}\;C_{coup}^{\prime \prime }\;$are coupling capacitors of, respectively, MC_1_ and MC_2_ windings, which are, structurally, capacitors with parasitic capacitances formed by a copper cylindrical electrode with coaxially located core elements and the first lower rows of, respectively, MC_1_ and MC_2_ windings.

In this case, the excitation voltage *u*_0_ = *U*_0max_·sin ω*t* (corresponding complex magnitude—${\dot{U}}_{0}$) is applied to MC_1_ and MC_2_ windings through, respectively, $C_{coup}^{\prime }\;\mathrm{and}\;C_{coup}^{\prime \prime }$, which are also electrical parameters of MC_1_ and MC_2_, and output signals ${\dot{U}}_{1}$ and ${\dot{U}}_{2}$ are read from respective input ends of MC_1_ and MC_2_ windings.

Assume that with a symmetrical MC_1_ and MC_2_ arrangement, $R_{1}=R_{2}=R;\;L_{1}=L_{2}=L;\;C_{1}=C_{2}=C;\;C_{coup}^{\prime }=C_{coup}^{"}=C_{coup}$. Then, considering that ${\dot{Z}}_{11}=R_{1}+j\cdot \omega \cdot L_{1}$ and ${\dot{Z}}_{21}=R_{2}+j\cdot \omega \cdot L_{2}$ are complex resistances of, respectively, MC_1_ and MC_2_; ${\dot{Z}}_{12}=-j/(\omega \cdot C_{1})$ and ${\dot{Z}}_{22}=-j/(\omega \cdot C_{2})$ are complex resistances of inter-turn capacitances of, respectively, MC_1_ and MC_2_; ${\dot{Z}}_{13}=-j/(\omega \cdot C_{coup}^{\prime })$ and ${\dot{Z}}_{23}=-j/(\omega \cdot C_{coup}^{"})$ are complex resistances of the coupling capacitors of, respectively, MC_1_ and MC_2,_ for the considered FS type, we will have:

(23) $${\dot{Z}}_{11}={\dot{Z}}_{21};\;{\dot{Z}}_{12}={\dot{Z}}_{22};{\dot{Z}}_{13}={\dot{Z}}_{23};\;\omega =\omega_{p}=1/\sqrt{L\cdot C_{cB}}$$

where $C_{coup}=0.56\cdot l/\ln(d_{2}/d_{1})$; *l* is the length of the overlapped part of the coupling capacitor plates; *d*_1_ and *d*_2_ are diameters of, respectively, the inner and outer coupling capacitor plates.

In the case when a TO made of a homogeneous structure or structural local uniformity material is in the working area of *FC_1_* and *FC_2_* and considering the aforementioned comments, for common-mode output signals ${\dot{U}}_{1}$ and ${\dot{U}}_{2}$ from, respectively, the output ends of measuring coils *MC*_1_ and *MC*_2_ (inductive elements of a bridge voltage divider), the condition ${\dot{U}}_{1}={\dot{U}}_{2}$ will always be met, and $\Delta {\dot{U}}_{\Sigma }={\dot{U}}_{1}-{\dot{U}}_{2}=0$.

**Figure 5:**
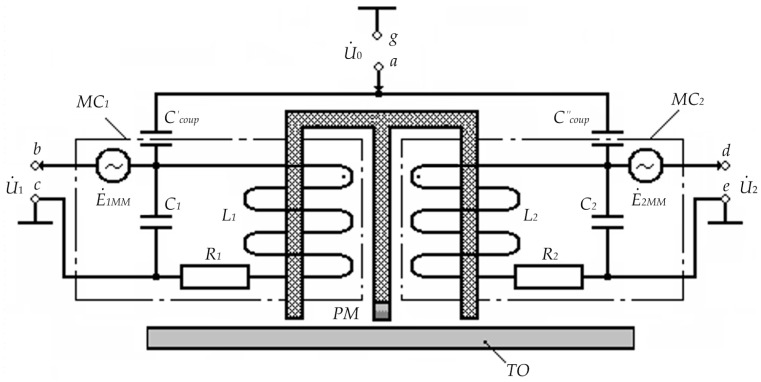
Circuit Diagram of the FS Substitution and Inclusion for the Third Mode.

2. The inductor mode, when the AC flow through windings of measuring coils MC_1_ and MC_2_ induces variable magnetic (excitation) fields in the material of the core’s FC_1_ and FC*_2_* elements.

3. The mode of μ-modulation due to the MM effect. Figure 5 shows the circuit diagram of the substitution and inclusion for the considered operating mode.

In this FS operating mode, the auxiliary magnetic fields induced in the ferrite cores of the FS half-elements modulate the magnetic permeability of these cores, which, in turn, accordingly variates the magnetic induction *B*_0_ of the PM magnetic field, inducing corresponding common-mode magnetic modulation EMFs ${\dot{E}}_{1MM}$ and ${\dot{E}}_{2MM}$ in measuring coils MC_1_ and MC_2_.

For the time-combined first, second, and third FS operating modes accompanied by corresponding physical effects, the difference between ${\dot{U}}_{1\Sigma }={\dot{U}}_{1}+{\dot{E}}_{1MM}$ and ${\dot{U}}_{2\Sigma }={\dot{U}}_{2}-{\dot{E}}_{2MM}$ is determined by the following equation:

(24) $$\Delta {\dot{U}}_{\Sigma }={\dot{U}}_{1\Sigma }-{\dot{U}}_{2\Sigma }=({\dot{U}}_{1}+{\dot{E}}_{1MM})-({\dot{U}}_{2}+{\dot{E}}_{2MM}).$$

For the case when a TO made of a material with a homogeneous structure is in the working area of FC_1_ and FC_2_ and considering the aforementioned comments, at the output ends of measuring coils MC_1_ and MC_2_, we will have ${\dot{E}}_{1MM}={\dot{E}}_{2MM}$, i.e., $\Delta {\dot{U}}_{\Sigma }=0$; when structural inhomogeneity occurs in the TO material in the working area of FC_1_ and FC_2_, ${\dot{E}}_{1MM}\neq {\dot{E}}_{2MM}$ and $\Delta {\dot{U}}_{\Sigma }\neq 0$, i.e., $\Delta {\dot{U}}_{\Sigma }=\Delta {\dot{E}}_{MM}$.

**Figure 6:**
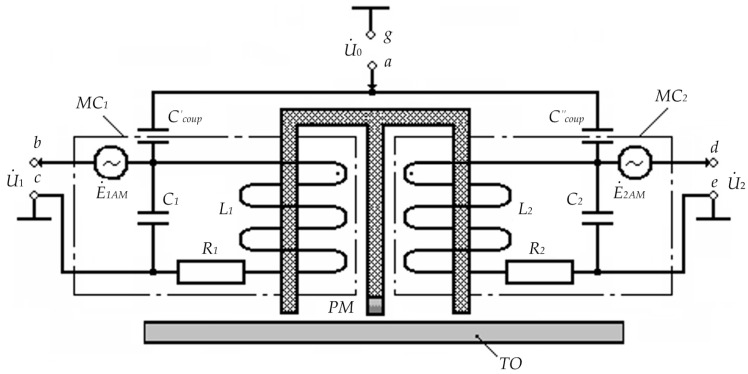
Circuit Diagram of the FS Substitution and Inclusion for the Fifth Mode.

4. The EMA converter mode. In this FS operating mode, the AC flow through windings of measuring coils MC_1_ and MC_2_ excites eddy currents on the surface of the conductive magnetic material, which, under a static magnetic field of the appropriate direction, are affected by forces transmitted subsequently to the crystal lattice through collisions and other interactions. As a reaction to such forces, spatially periodic acoustic waves arise in the structure of the conductive magnetic material.

5. The mode of μ-modulation due to the EMA effect. Figure 6 shows the circuit diagram of the proposed FS version substitution and inclusion for the considered operating mode.

In this case, spatially periodic acoustic waves mechanically affect the domain system of the conductive magnetic material structure of the core’s FC_1_ and FC_2_ elements, thereby modulating the magnetic permeability of their material. Herewith, it should be noted that the resulting mechanical stresses significantly affect the magnetization state.

The resulting variations in the magnetic induction *B*_0_ of the PM magnetic field induce the corresponding common mode relative to the ‘case’ acoustic modulation EMFs ${\dot{E}}_{1AM}$ and ${\dot{E}}_{2AM}$ in measuring coils MC_1_ and MC_2_.

For the time-combined first, fourth, and fifth FS operating modes accompanied by corresponding physical effects, the difference between ${\dot{U}}_{1\Sigma }={\dot{U}}_{1}+{\dot{E}}_{1AM}$ and ${\dot{U}}_{2\Sigma }={\dot{U}}_{2}+{\dot{E}}_{2AM}$ can be expressed as follows:

(25) $$\Delta {\dot{U}}_{\Sigma }={\dot{U}}_{1\Sigma }-{\dot{U}}_{2\Sigma }=({\dot{U}}_{1}+{\dot{E}}_{1AM})-({\dot{U}}_{2}+{\dot{E}}_{2}{}_{AM}).$$

For the case when a TO made of a material with a homogeneous structure is in the working area of FC_1_ and FC_2_ and considering the aforementioned comments, at the output ends of measuring coils MC_1_ and MC_2_, we will have ${\dot{E}}_{1AM}={\dot{E}}_{2AM}$, i.e., $\Delta {\dot{U}}_{\Sigma }=0$.

When structural inhomogeneity occurs in the TO material in the working area of FC_1_ and FC_2_, ${\dot{E}}_{1AM}\neq {\dot{E}}_{2AM}$ and $\Delta {\dot{U}}_{\Sigma }\neq 0$, i.e.,

(26) $$\Delta {\dot{U}}_{\Sigma }=\Delta {\dot{E}}_{AM}$$

For all the five time-combined FS operating modes, we can write an equation:

(27) $$\Delta {\dot{U}}_{\Sigma }={\dot{U}}_{1\Sigma }-{\dot{U}}_{2\Sigma }=({\dot{U}}_{1}+{\dot{E}}_{1AM}+{\dot{E}}_{1MM})-({\dot{U}}_{2}+{\dot{E}}_{2AM}+{\dot{E}}_{2MM})$$

For the case when a TO made of a material with a homogeneous structure is in the working area of FC_1_ and FC*_2_* and considering the aforementioned comments, at the output ends of measuring coils MC_1_ and MC_2_, we will have ${\dot{U}}_{1}={\dot{U}}_{2}$, ${\dot{E}}_{1AM}={\dot{E}}_{2AM}$, and ${\dot{E}}_{1MM}={\dot{E}}_{2MM}$, i.e., $\Delta {\dot{U}}_{\Sigma }=0$.

When structural inhomogeneity occurs in the TO material in the working area of FC_1_ and FC_2_, ${\dot{U}}_{1}={\dot{U}}_{2}$,${\dot{E}}_{1AM}\neq {\dot{E}}_{2AM}$, ${\dot{E}}_{1MM}\neq {\dot{E}}_{2MM}$, and $\Delta {\dot{U}}_{\Sigma }\neq 0$, i.e.,

(28) $$\Delta {\dot{U}}_{\Sigma }=\Delta {\dot{E}}_{AM}+\Delta {\dot{E}}_{MM}$$

where $\Delta {\dot{E}}_{AM}={\dot{E}}_{1AM}-{\dot{E}}_{2AM}$; $\Delta {\dot{E}}_{MM}={\dot{E}}_{1MM}-{\dot{E}}_{2MM}$.

The analysis of Equation (28) shows that when structural inhomogeneity (flaw) occurs in the material in the working area of FC_1_ and FC_2_, the difference signal from the MC_1_ and MC_2_ outputs will be determined by two major realizable physical effects (EMA and MM), which allows for detecting the structural inhomogeneity in the TO material reliably.

## 5. Generalized Physical FS Diagram

Like any other technical device, the proposed FS version is a complex hierarchical system characterized by many structural elements and links between them. Such a system operation is based on the manifestation of many interrelated physical effects, the totality of which is, in fact, the FS physical diagram.

**Figure 7:**
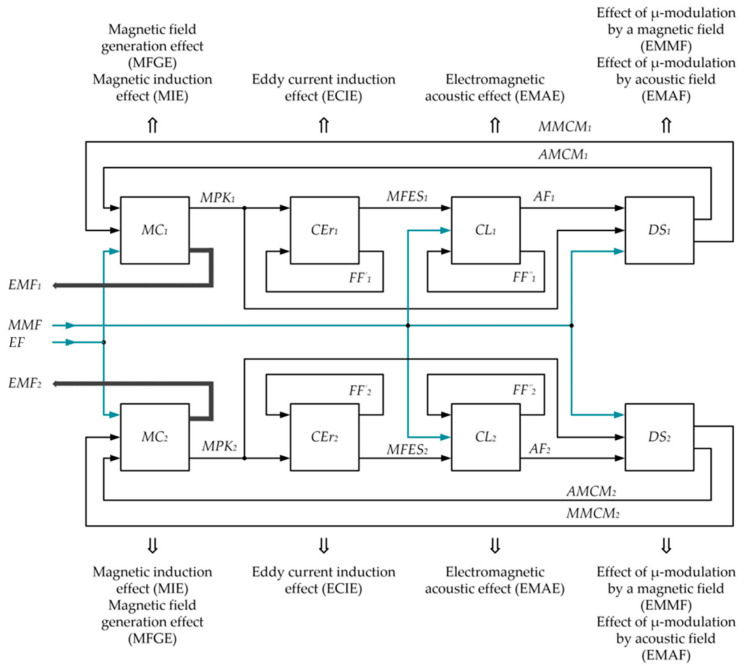
The FS Physical Diagram.

Figure 7 shows the generalized physical diagram underlying the operation of the proposed FS version.

In Figure 7: MC_1_ and MC_2_ are measuring coils of, respectively, FC-I and FC-II; CEr1 and CEr2 are conduction electrons of the ferromagnetic material of, respectively, the core elements FC-I and FC-II; CL_1_ and CL_2_ are crystal lattices of the material of, respectively, the core elements FC-I and FC-II; DS_1_ and DS_2_ are domain systems of the material of, respectively, the core elements FC-I and FC-II; EMF_1_ and EMF_2_ are electromotive forces of induction of, respectively, MC_1_ and MC_2_; MMF is the magnet’s magnetic field; EF is the alternating electric field induced between CE_1_ and CE_2_ and the lower wire layer of windings of, respectively, MC_1_ and MC_2_; MPK_1_ and MPK_2_ are magnetic fields induced by MC_1_ and MC_2_ excitation currents in the material of, respectively, the core elements FC-I and FC-II; FF′_1_, FF^″^_1_ and FF_′__2_, FF^″^_2_ are force fields of different levels, manifested as physical effects in the crystal lattices of the material of the MP elements, respectively, FC-I and FC-II; MFEC_1_ and MFES_2_ are magnetic fields induced by eddy currents in the material of, respectively, the core elements FC-I and FC-II; AF_1_ and AF_2_ are acoustic fields excited in the crystalline lattices of the material of, respectively, the core elements FC-I and FC-II; MMCM_1_ and MMCM_2_ are magnetically modulated PM fields in, respectively, FC-I and FC-II; AMCM_1_ and AMCM_2_ are acoustically modulated PM fields in, respectively, FC-I and FC-II. This physical FS diagram provides a complete picture of which given input parameters are converted into given output parameters, i.e., reflects the FS working function, and diagrams of individual physical effects describe the FS physical basis and the functional relationship between its structural elements.

The analysis of possible physical effects and aforementioned analytical Equations (19), (20), (22) and (28) proves the soundness of the proposed ideas, implemented in the format of the considered technical solution and determining the design features of its practical implementation.

## 6. Experimental Studies

To prove the soundness of the proposed technical solutions, the authors performed appropriate experimental studies, where the flaw detectability index was chosen to estimate the NDT reliability.

The relative comparison technique was used to determine the flaw detectability index in respect of the tested NDT system. It was based on comparing the results of testing performed by this system with those obtained by the reference NDT system.

In this case, the tested NDT system was a fluxgate sensor with bifactor excitation, and the DF-105 flaw detector-gradiometer comprising an electronic unit and an FP-4 fluxgate converter, interconnected by a flexible cable, was chosen as the reference NDT system. FP-4 functionally converted the magnetic field strength gradient into an electrical signal, amplified and processed accordingly, and the final result was read on the LC display DF-201.1.

Due to objective organizational and technological difficulties, two limited subsets of reference samples (RS) simulating subsurface flaws were used in the experimental study, which had the form of ferrite prismatic bars of the same size with dimensions (50 mm × 20 mm × 10 mm), side cylindrical holes of a certain diameter, and the appropriate depth of location, made of nickel-zinc alloy 600HH.

**Figure 8:**
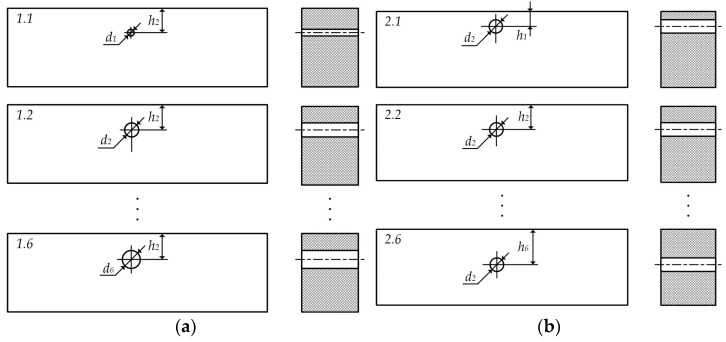
RS Subset with Subsurface Flaws: First Subset (**a**) and the Second RS Subset (**b**).

The first subset, used at the first experimental stage, consisted of six ferrite prismatic bars with side cylindrical holes of different diameters (*d*_1_, *d*_2_, …, *d*_6_), located in the corresponding RSs at the same depth (*h*_2_) (Figure 8a).

The second RS subset, used at the second experimental stage, consisted of six ferrite prismatic bars with side cylindrical holes of the same diameter (*d*_2_) located in the corresponding RSs at different depths (*h*_1_, *h*_2_, …, *h*_6_) (Figure 8b).

The dimensions of the RS flaws in the form of side cylindrical holes and their probable depths are given in Table 1.

In experimental studies, two identical RS sets were used, consisting of the first and second RS subsets each. The first RS set was pre-magnetized by an attachable magnetizing device containing constant magnets. This attachable magnetizing device generated a magnetic induction with a strength of 25 mT in the reference sample material. The first set of RS subsets was used in the NDT performed by the reference NDT system, and the second RS set, consisting of the already non-magnetized first and second RS subsets of EO, was used in the NDT performed by the tested NDT system.

**Figure 9:**
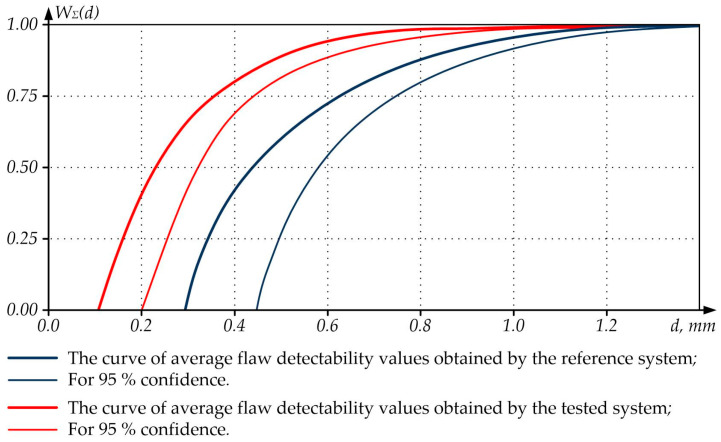
Curves of Flaw Detectability Depending on the Flaw Diameter.

**Figure 10:**
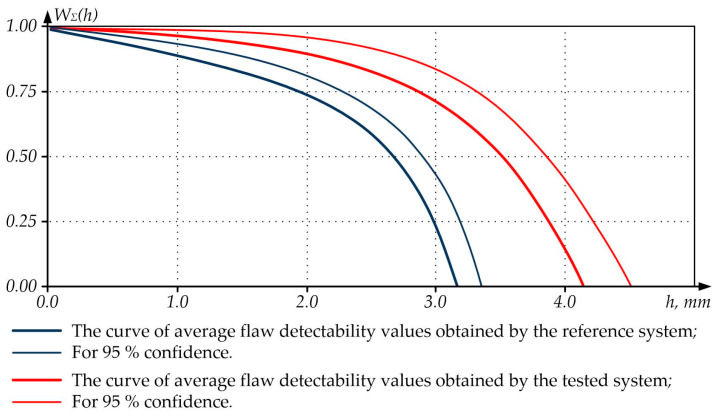
Curves of Flaw Detectability Depending on the Flaw Depth.

Based on the results of experimental data processing according to the appropriate procedure (GOST R 50.04.07-2018), flaw detectability curves (POD curves, Probability of Detection) were built to describe the distribution of the flaw detection probability by the flaw size (Figure 9 and Figure 10) and reflect the opportunities of the reference and tested NDT systems. Note that POD curves also allow for forecasting the detectability of flaws of various sizes in an object due to the known functional dependence of the flaw detection probability on the flaw size. The use of POD curves to assess the quality of the control of the tested NDT systems is based on the correlation between the POD values for various groups of flaws, size, and form changes. This makes it easier to compare such POD values and allows for using a smaller number of flaws, forms, and size changes during tests in contrast to the comparison conducted for defect groups that were assessed without building POD curves.

The analysis of Figure 9 shows that the tested NDT system has increased sensitivity (the size of the smallest detectable flaw has noticeably decreased) and a reduced confidence interval compared to the reference one, which indicates an increase in the tested NDT system’s resolution in respect of the flaws.

The analysis of Figure 10 allows for drawing the conclusion that the tested NDT system can detect flaws at a greater depth and with an increased confidence interval compared to the reference one, which indicates that the tested NDT system is more reliable in determining the flaw depth.

**Figure 11:**
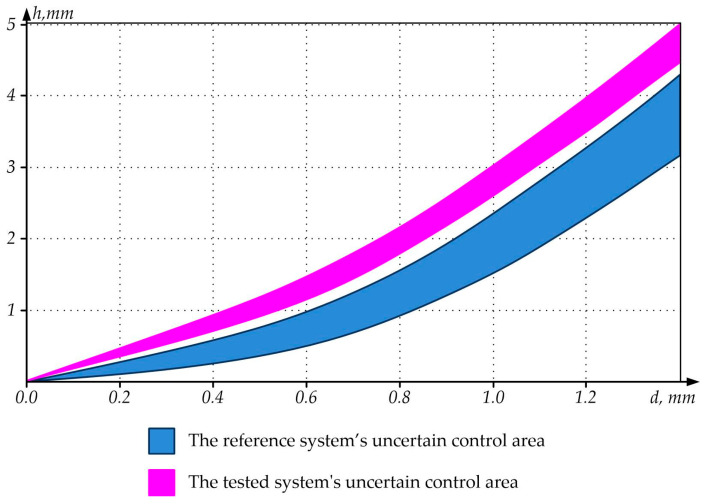
Flaw Detectability Areas of the Reference and Proposed Systems.

For the sake of clarity, the dependencies of the detection of flaws in the form of side cylindrical holes on their diameter *d* and depth *h* were also plotted based on the experimental study results (Figure 11).

Figure 11 shows that the tested NDT system has a narrower uncertain control area and can record smaller flaws at greater depths compared to the reference NDT system.

Note that this study is the first stage of developing a new type of fluxgate flaw detector. This stage was aimed at developing and theoretically justifying the possibility of the fluxgate sensor’s bifactor excitation mode. The second stage of the study will involve developing a hardware implementation of the flaw detector proposed, its extended experimental study, and a detailed comparison of its performance and opportunities with the available similar-purpose equipment.

## 7. Conclusions

Summarizing, one can state that, compared to the conventional version of the fluxgate with magnetic excitation, the proposed excitation technique based on the bifactor excitation mode has the following advantages:

1. A significantly simpler design as MC_1_ and MC_2_ combine two functions such as generating a variable magnetic field of excitation and reading the data signal;
2. Increased noise immunity due to the use of MC_1_ and MC_2_ as arms of a capacitive-inductive measuring bridge with a difference signal of its measuring diagonal (compensation of common-mode noise components and the temperature drift consequences);
3. Increased sensitivity without deterioration of the conversion accuracy, ensured by a set of properties of the material of the core’s FC-I and FC-II structural elements, manifested as various physical effects when exposed to resonant physical fields;
4. Reduced power consumption.

The aforementioned advantages of the proposed FS version compared to available prototypes demonstrate the real prospects for its widest commercial application to solve various control and diagnostics problems.

The proposed new fluxgate excitation technique and the variant of its technical implementation also open real prospects for more detailed research in the field of complex applied use of various μ-modulation types by magnetic equipment developers, the results of which can be effectively used in developing various fluxgate versions based on new physical operation principles.

## Figures and Tables

**Table 1:** The dimensions of the RS flaws.

I	1	2	3	4	5	6
*d_i_*, mm	0.1	0.3	0.5	0.8	1.1	1.4
*h_i_*, mm	0.5	1.5	2.5	3.5	4.0	4.5

## References

[1] Ishaya Z.D., Dauda M., Pam G.Y., Kulla D.M. (2013). Destructive testing and production system integrity. Adv. Appl. Sci. Res..

[2] Doktor M.S., Fox C., Kurz W., Stockis J.-P. (2018). Characterization of steel buildings by means of non-destructive testing methods. J. Math. Ind..

[3] Raj B., Jürgen Buschow K.H., Flemings M.C. (2001). Nondestructive Testing and Evaluation: Overview. Encyclopedia of Materials: Science and Technology.

[4] Mix P.E. (2005). Introduction to Nondestructive Testing.

[5] Paipetis A.S., Matikas T.E., Aggelis D.G., Van Hemelrijck D. (2012). Emerging Technologies in Non-Destructive Testing V.

[6] Omar M. (2012). Nondestructive Testing Methods and New Applications.

[7] Ficili F. (2012). Non-Destructive Testing by Magnetic Techniques.

[8] Stefanita C.G. (2012). Magnetic Nondestructive Testing Techniques. Magnetism.

[9] Cheng W. (2017). Electromagnetic nondestructive evaluation of defects in ferromagnetic structures. AIP Conf. Proc..

[10] Jiles D.C. (2001). Magnetic methods in nondestructive testing. Encyclopedia of Materials Science and Technology.

[11] Tomáš I. (2004). Non-Destructive Magnetic Adaptive Testing of Ferromagnetic Materials. J. Magn. Magn. Mater..

[12] Liu S., Sun Y., Jiang X., Kang Y. (2020). A Review of Wire Rope Detection Methods, Sensors and Signal Processing Techniques. J. Nondestruct. Eval..

[13] Zhou P., Zhou G.B., Zhu Z.C., He Z.Z., Ding X., Tang C.Q. (2019). A review of non-destructive damage detection methods for steel wire ropes. Appl. Sci..

[14] Bryakin I.V., Bochkarev I.V., Khramshin R.R. The Power Cables Quality Diagnostics. Proceedings of the International Russian Automation Conference (RusAutoCon).

[15] Yigzew F.E., Kim H.S., Park S. Non-Destructive Damage Detection for Steel Pipes Using MFL Based 3D Imaging Method. Proceedings of the 11th International Symposium on Steel Structures.

[16] Xionga E., Zhao N., Yan Z., Yang L., He H. (2016). Magnetic Nondestructive Testing Techniques of Constructional Steel. MATEC Web Conf..

[17] Chady T., Grochowalski J.M. (2019). Eddy current transducer with rotating permanent magnets to test planar conducting plates. Sensors.

[18] Ripka P. (2000). Magnetic Sensors and Magnetometers.

[19] Lenz J.E., Edelstein A.S. (2006). Magnetic Sensors and Their Applications. IEEE Sens. J..

[20] Tumanski S. (2013). Modern magnetic field sensors—A review. Prz. Elektrotechniczn.

[21] Ripka P., Janosek M. (2010). Advances in Magnetic Field Sensors. IEEE Sens. J..

[22] Can H., Topal U. (2015). Design of Ring Core Fluxgate Magnetometer as Attitude Control Sensor for Low and High Orbit Satellites. J. Supercond. Nov. Magn..

[23] Repelianto A.S., Kasai N. (2019). The Improvement of Flaw Detection by the Configuration of Uniform Eddy Current Probes. Sensors.

[24] Nagendran R., Mohanty I., Thanikai Arasu A.V., Baskaran R. (2018). Transient Eddy Current NDE System Based on Fluxgate Sensor for the Detection of Defects in Multilayered Conducting Material. J. Nondestruct. Eval..

[25] Bryakin I.V., Bochkarev I.V., Khramshin R.R. Two-Axis Fluxgate Magnetometer with a New Principle of Excitation. Proceedings of the International Russian Automation Conference (RusAutoCon).

[26] Bochkarev I.V., Bryakin I.V., Khramshin V.R. Ferroprobe Magnetometer with Preset Excitation Field Induction Mode. Proceedings of the International Ural Conference on Electrical Power Engineering (UralCon).

[27] Bryakin I.V., Bochkarev I.V. (2022). Method for excitation of ferroprobes and modulator apparatus for implementation thereof. RF Patent.

[28] Faria J.A.B. (2008). Electromagnetic Foundations of Electrical Engineering.

[29] Morris A.S., Langari R. (2015). Measurement and Instrumentation. Theory and Application.

[30] Woan G. (2003). The Cambridge Handbook of Physics Formulas.

[31] Morrish A.H. (2001). The Physical Principles of Magnetism.

